# Trichomonosis, a Common Curable STI, and Prostate Carcinogenesis—A Proposed Molecular Mechanism

**DOI:** 10.1371/journal.ppat.1002801

**Published:** 2012-08-09

**Authors:** Siobhan Sutcliffe, Calvin Neace, Nancy S. Magnuson, Raymond Reeves, J. F. Alderete

**Affiliations:** 1 Division of Public Health Sciences and the Alvin J. Siteman Cancer Center, Department of Surgery, Washington University School of Medicine, St. Louis, Missouri, United States of America; 2 School of Molecular Biosciences, College of Veterinary Medicine, Washington State University, Pullman, Washington, United States of America; Duke University Medical Center, United States of America

## Why Study Trichomonosis and Prostate Cancer?

Trichomonosis, a sexually transmitted infection (STI) caused by the protist *Trichomonas vaginalis*, has significant public health relevance. The annual incidence is ∼8 million women in the United States and 170 million worldwide, with an equal number of infected male partners [1]. Both men and women infected with *T. vaginalis* are at increased risk for human immunodeficiency virus infection [1]. Recent evidence suggests this STI is associated with increased risk of prostate cancer, the most commonly diagnosed cancer and the second leading cause of cancer death among men in the United States [2]. There is no immunity to *T. vaginalis*, and a hallmark of this STI agent is persistence. Most *T. vaginalis* infections in men are asymptomatic, and few are diagnosed and treated; thus, infections persist. In older, pre-antibiotic era studies, *T. vaginalis* was frequently found in prostate fluid specimens from asymptomatic male partners of women with trichomonosis, leading to the belief that the prostate might serve as the reservoir for trichomonosis in men [3]. Trichomonosis may cause chronic prostatitis, and researchers have identified trichomonads in the prostatic urethra, glandular lumina, submucosa, and stroma [4] and, more recently, in benign hyperplastic prostatic tissue [3]. They also observed foci of nonspecific acute and chronic inflammation, as well as intraepithelial vacuolization, near trichomonads, leading them to propose that trichomonosis might contribute to prostate carcinogenesis [4].

## What Epidemiologic Evidence Links Trichomonosis to Prostate Cancer?

The α-actinin protein is one of the most immunogenic proteins of *T. vaginalis*. This protein is not found among other microorganisms and shares little amino acid sequence identity with its human homolog. Among *Tritrichomonas suis*, *Candida albicans*, and *Saccharomyces cerevisiae*, this protein has only 4.8%, 9.8%, and 11.1% overall identities, respectively. *H. sapiens* homologs of α-actinin have only 25% overall identity. Further, we performed epitope mapping using as probes representative sera of *T. vaginalis*–infected female and male patients that were highly reactive to both whole cell *T. vaginalis* and α-actinin by ELISAs [5]–[7]. Surprisingly, the female sera reacted with 13 epitopes scattered throughout the entire α-actinin protein, whereas male sera detected only 5 epitopes that were identical to a subset of those recognized by the female sera. These epitopes have no identity to other proteins in databanks. This indicates that the female and male antibody responses to α-actinin are polyclonal and recognize multiple epitopes. There is no detection using these female and male sera, singly or in combination, with purified human α-actinin protein. Therefore, a highly seropositive reaction to this protein in humans indicates exposure to *T. vaginalis*.

Recently, in a number of independent studies, trichomonosis history, as measured by serum antibodies against *T. vaginalis* α-actinin protein, was found to be associated with prostate cancer risk. In a large nested case-control study within the Health Professionals Follow-up Study, a positive relation between *T. vaginalis* serostatus and prostate cancer risk was found [6]. This association was slightly stronger for high-grade disease. This study was followed by analyzing two additional populations, the Prostate Cancer Prevention Trial (PCPT) [7] and the Physicians' Health Study (PHS) [5]. While the PCPT study did not observe an association, the PHS study detected significant positive associations for extraprostatic and fatal prostate cancer [5]. It was noted that the PCPT null findings may have been due to the very early stage of prostate cancer examined in the trial. Finally, as further epidemiologic evidence for an association between trichomonosis and prostate cancer risk, African Americans, who have the highest incidence of trichomonosis [1], also have the highest risks of prostate cancer diagnosis and death [2]. Thus, an accumulating body of evidence suggests that trichomonosis contributes to prostate carcinogenesis, a particularly more aggressive or fatal disease.

## What Is a Possible Molecular Mechanism for *T. vaginalis*–Mediated Prostate Carcinogenesis?

Despite the observed relation between *T. vaginalis* seropositivity and prostate cancer risk, there is a dearth of knowledge regarding how this parasite might contribute to prostate carcinogenesis. We propose two synergistic molecular mechanisms. First, we hypothesize that *T. vaginalis* infection may contribute to carcinogenesis via inflammation, which is believed to be important for prostate cancer development [8]. Second, we hypothesize and present preliminary data and published reports that *T. vaginalis* adherence or binding of specific trichomonad adhesin proteins to normal prostate epithelial cells (PECs) triggers a cell-signaling cascade through known proto-oncogenes, *PIM1*, *c-MYC*, and *HMGA1*, that may ultimately lead to prostate carcinogenesis [9]–[12].

## Does *T. vaginalis* Mediate Inflammation?

*T. vaginalis* infection is characterized by cytopathogenicity, and an influx of leukocytes and chronic inflammation [1]. Parasite adherence to vaginal epithelial cells (VECs) induces expression of monocyte chemoattractant protein-1 and IL-8, pro-inflammatory cytokines involved in neutrophil recruitment [13]. High levels of IL-8, leukotreine B_4_, and neutrophils have been found in vaginal secretions from patients with trichomonosis [14]. Neutrophils may contribute to carcinogenesis by secreting a variety of oxygen- and nitrogen-based reactive molecules capable of damaging DNA and nearby cells [15]. *T. vaginalis* attachment to VECs has also been shown to lead to elevated levels of IL-6 [16], a key inflammatory mediator associated with worse prostate cancer presentation/prognosis [17] and with prostate cancer incidence and mortality among healthy-weight men in a large prospective study [18]. More recently, we have shown that parasite contact with PECs induces expression of IL-6 (unpublished data). The key point is that *T. vaginalis* infection promotes synthesis of pro-inflammatory cytokines that may be important in prostate carcinogenesis.

## What of the PIM1-HMGA1-COX2 Cell-Signaling Cascade?

### PIM1

**Figure 1:**
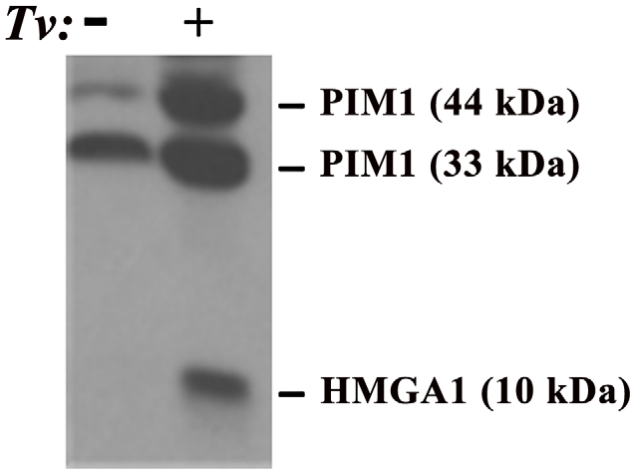
Demonstration of elevated amounts of PIM1 and HMGA1 proteins in PECs after adherence by *T. vaginalis* (*Tv*). In this experiment, trichomonads were added to T25 confluent monolayers of PECs (lane labeled+) using a parasite to PEC ratio of 10∶1. PECs without added organisms are labeled with a minus sign (−). This ratio of 10∶1 was chosen because it has been shown to yield at least one parasite attached per epithelial cell in adherence assays and to optimally signal epithelial cells for up-regulation of expression of genes [13], [27]. After visible attachment to PECs, non-adherent organisms were decanted and fresh PEC medium added followed by incubation at 37°C for an additional 30 min. The flask was then placed directly in ice for detachment of organisms, after which PECs were washed and removed from the flask for preparation of total proteins for immunoblotting using established protocols, polyclonal rabbit antibodies produced in our laboratories, and equal loading of protein onto gels, as detailed previously [28]. Under conditions of exposure of PECs with or without *T. vaginalis*, no change in the amount of other cellular proteins was detected, as evidenced by no changes in the amounts of Akt and Bad, and this served to show equal amounts of total proteins loaded onto gels for SDS-PAGE and immunoblotting [28]. Prebleed rabbit serum was used as the negative control and gave no reactivity.

The *PIM1* gene is a known proto-oncogene [19] whose encoded protein belongs to a small family of serine/threonine kinases that are unique because they are constitutively active. PIM1 is believed to be important for carcinogenesis because expression of this gene can lead to genomic instability and the preservation of potentially cancer-producing genomic alterations by promoting cell survival under conditions in which these alterations would not be normally tolerated [20]. PIM1 may be important for prostate cancer, in particular because altered levels of PIM1 were observed in a study comparing malignant-to-benign prostate specimens by gene expression microarray and clinically stratified prostate cancer specimens by protein arrays [15], as well as in studies comparing malignant-to-benign prostate specimens and studies of prostate cancer tissue and cell lines using immuno-histochemistry with antibody to PIM1 [11]. Of particular relevance for *T. vaginalis* infection, we have demonstrated that *T. vaginalis* contact leads to increased PIM1 expression in PECs (see below and Figure 1), providing a possible molecular mechanism by which *T. vaginalis* contributes to prostate carcinogenesis. Importantly, it has been demonstrated that *PIM1* gene expression is induced by IL-6 via the JAK/STAT signaling pathway [21] and that *T. vaginalis*–induced IL-6 likely provides an additional molecular link between exposure of human PECs to *T. vaginalis*, stress, and cancer induction.

### HMGA1

**Figure 2:**
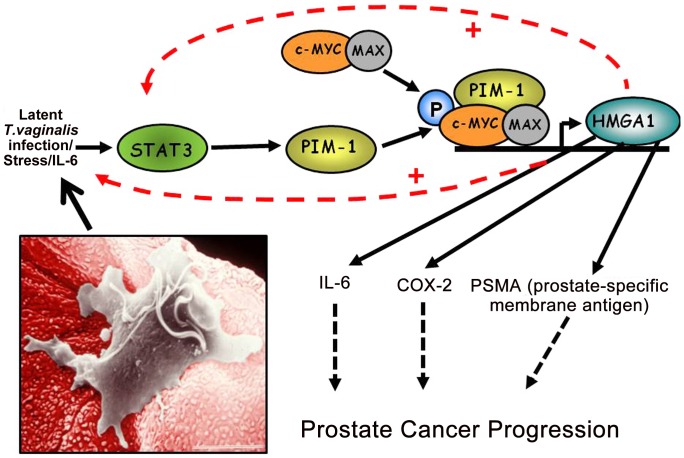
A working model of how chronic, latent *T. vaginalis* infection of prostate tissue up-regulates the signaling cascade leading to prostate carcinogenesis. Production of IL-6 leads to transcriptional activation of the STAT3-PIM1-HMGA1 cascade. In this case induced transcriptional activation of the *HMGA1* proto-oncogene contributes directly to prostate cancer progression via pathways involving COX2 and the prostate-specific membrane antigen. The inset shows a *T. vaginalis* organism adherent to a VEC, and the same mechanism of cytoadherence occurs for PECs.

The proto-oncogene *HMGA1* encodes a chromatin “architectural transcription factor” [22] that, evidence indicates, acts downstream of PIM1 in an HMGA1-mediated prostate cancer induction pathway (Figure 2). Phosphorylation of c-MYC by the PIM1 kinase has been shown to induce binding of a PIM1/c-MYC complex to the E box in the promoter of c-MYC target genes, such as *HMGA1*, and induce their transcription [23]. Both c-MYC and HMGA1 have also been found to be over-expressed in prostate cancers, suggesting the importance of the PIM1/c-MYC/HMGA1 signaling cascade in prostate carcinogenesis. Further evidence supporting a role for HMGA1 in prostate carcinogenesis includes the observation that high levels of HMGA1 in prostate cancer cells are related to enhanced proliferation and metastasis in vivo, studies suggesting that HMGA1 is involved in prostate cell chromosomal instability and rearrangements, and the fact that HMGA1 regulates transcription of a number of genes involved in various aspects of cell transformation and metastatic tumor progression. For example, the Prostate-Specific Membrane Antigen (PSMA) gene promoter has binding sites for HMGA1, and experiments have shown that transcription of both the human *STAT3* [24] and *COX-2* [25] genes are regulated in vivo by binding of the HMGA1 protein to their promoter regions. The oncogenic effects of HMGA1 may be further amplified because activation of the *STAT3* gene promoter by HMGA1 (Figure 2, dashed line) may potentially lead to a chronic self-reinforcing “feed-back” stimulatory loop resulting in constitutively high levels of HMGA1 expression. As for PIM1, HMGA1 may also be important for *T. vaginalis*–mediated prostate carcinogenesis as part of the PIM1→c-MYC→HMGA1 signaling cascade because we have recently demonstrated that *T. vaginalis* contact up-regulates expression of HMGA1 in PECs (Figure 1). *HMGA1* transcription is also known to be induced by cell stress and IL-6-mediated inflammation, possibly providing a further mechanism by which *T. vaginalis* may contribute to prostate carcinogenesis. As we have also shown that *T. vaginalis* induces IL-6 in PECs (unpublished data), we hypothesize that this stimulation is, likewise, due to the downstream binding of HMGA1 to the *IL-6* promoter that has binding sites for the protein, thereby providing increased IL-6 expression, which can then reinforce the cascade and contribute to prostate cancer progression by a separate, related mechanism.

### COX-2

We believe that COX-2 may be involved in *T. vaginalis*–mediated prostate carcinogenesis because we have recently shown that *COX-2* expression is induced in primary human VECs upon interaction with either intact *T. vaginalis* or purified trichomonad adhesin AP65 [13]. Further, HMGA1 up-regulates expression of *COX-2* [25], and COX-2 over-expression in prostate cancer has been documented and associated with both cancer initiation and progression. Like HMGA1, COX-2 over-expression has also been demonstrated in many other cancers, including breast, colorectal, head and neck, esophageal and non-small-cell lung cancers, and prostatic hyperplasia. COX-2 may play a role in cancer initiation and progression by affecting cell proliferation, mitosis, cell adhesion, apoptosis and immune surveillance, and/or angiogenesis. Interestingly, polyamines also regulate levels of COX-2 in human airway epithelial cells [26], and *T. vaginalis* secrete large amounts of polyamines (putrescine) during growth [27]. Polyamines play a role in cell cycle regulation in various types of cancers. The secreted polyamines, in concert with the induced host genes, might alter cell cycle regulation and result in a proliferative phenotype. Thus, transcriptional activation of the *HMGA1* proto-oncogene promotes prostate cancer progression via pathways that involve both COX2 and PSMA.

In summary, although several epidemiologic studies have observed positive associations between *T. vaginalis* seropositivity and prostate cancer risk, a major knowledge gap exists in understanding how *T. vaginalis* activates signal transduction pathways known to be associated with prostate carcinogenesis. We present testable hypotheses and a working model (Figure 2) supported by evidence [19] and believe that future research should investigate these novel findings at the molecular level.
